# Supplementary figures and images for: Global biochemical analysis of plasma, serum and whole blood collected using various anticoagulant additives
Source: PLoS One. 2021 Apr 8;16(4):e0249797. doi: 10.1371/journal.pone.0249797 (PMC8031419; doi:10.1371/journal.pone.0249797)

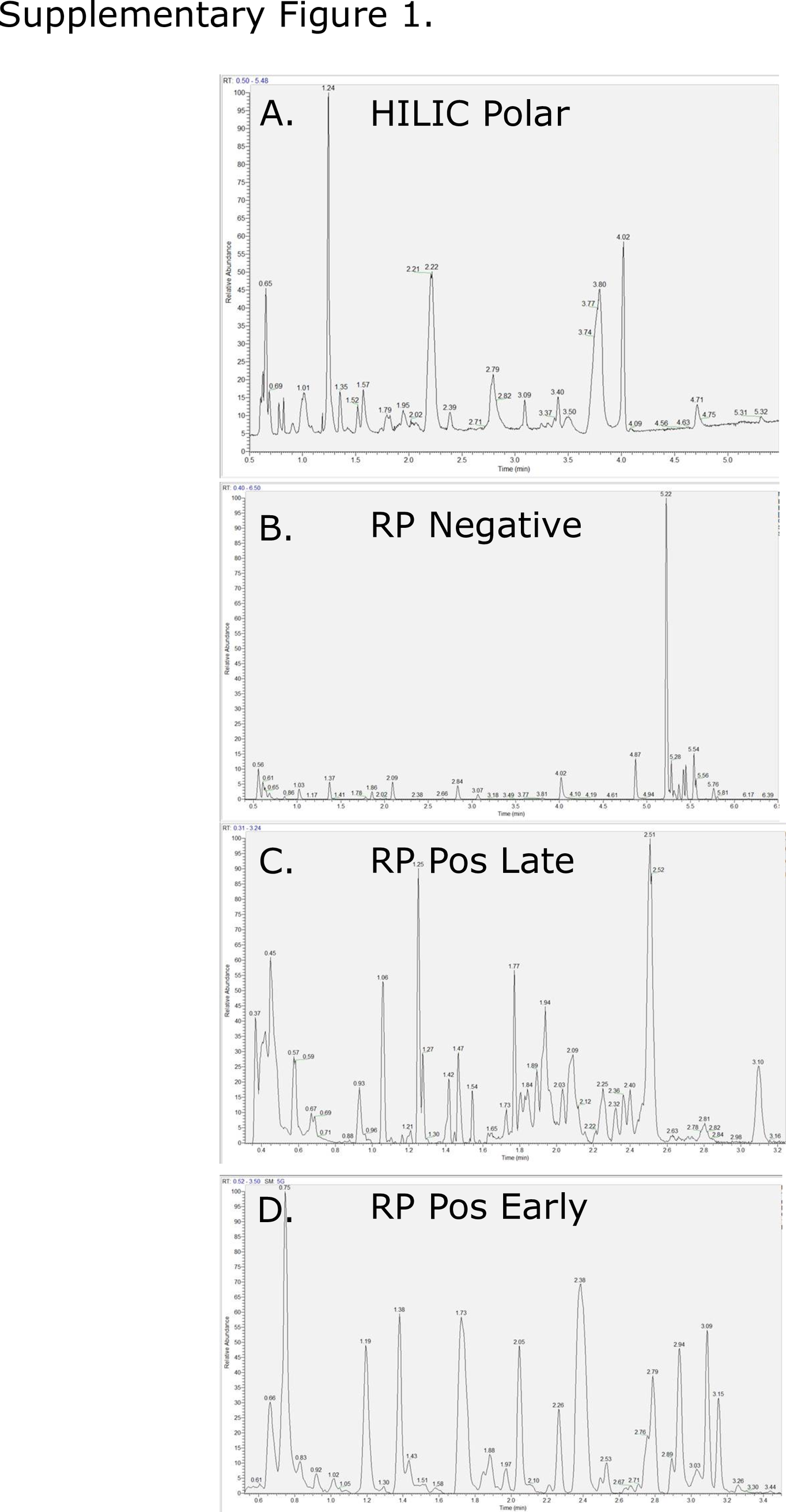

Supplement: S1 Fig — A) HILIC Polar method, B) LC-MS/MS Negative, C) LC-MS/MS Positive Late, and D) LC-MS/MS Positive Early. (TIF) [file pone.0249797.s001.tif]

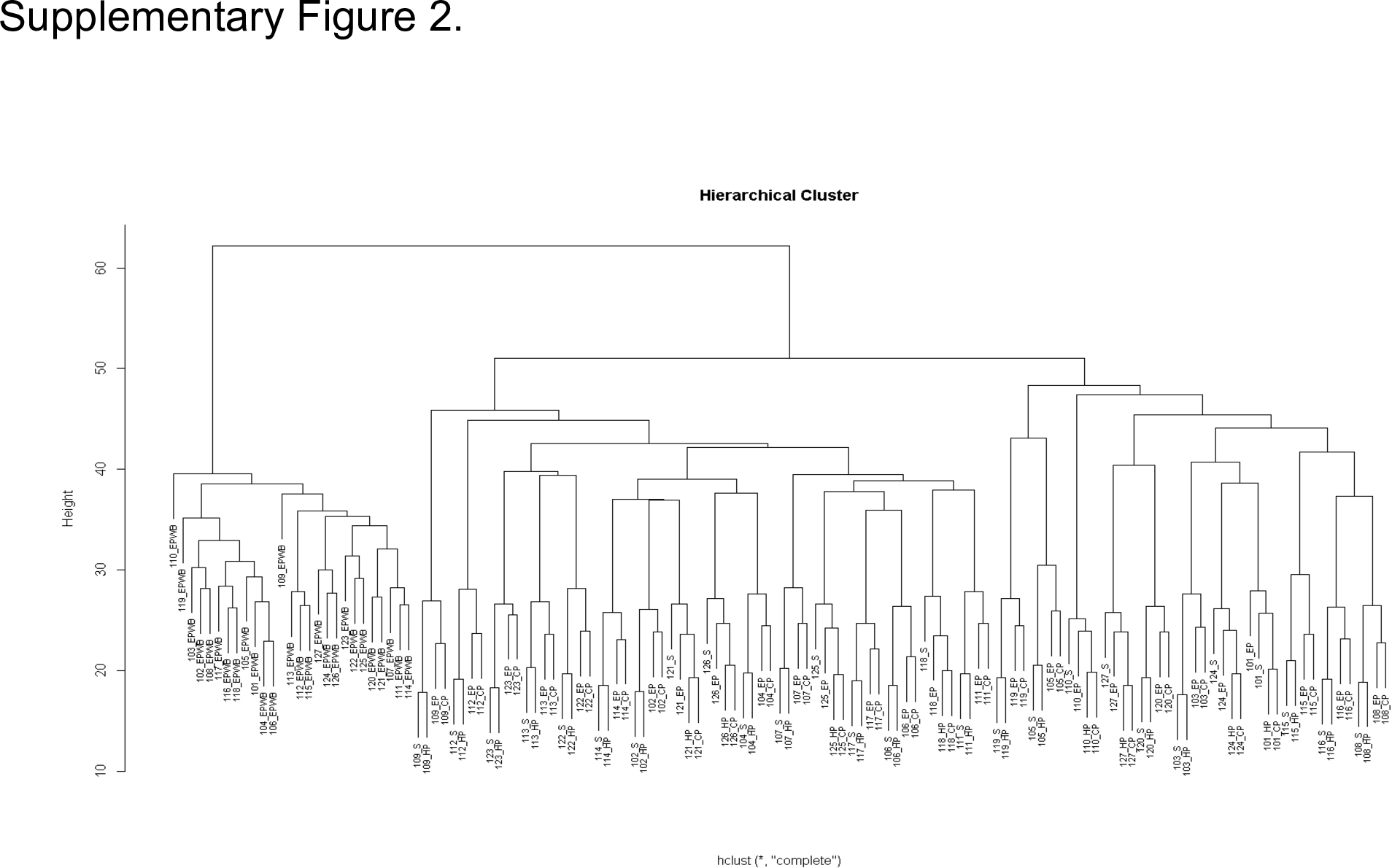

Supplement: S2 Fig — CP—citrate plasma; HP—heparinized plasma; S—serum; EP—EDTA plasma; EPWB—EDTA anticoagulated whole blood. The three digit codes indicate the blinded donor number for the study. (TIF) [file pone.0249797.s002.tif]
