# Supplementary material for: Global biochemical analysis of plasma, serum and whole blood collected using various anticoagulant additives
Source: PLoS One. 2021 Apr 8;16(4):e0249797. doi: 10.1371/journal.pone.0249797 (PMC8031419; doi:10.1371/journal.pone.0249797)
Supplement: S2 Table — (DOCX) [file pone.0249797.s004.docx]

S2 Table. Summary of statistical differences between sample types.

|  | Direction of Change | Range (Fold of change) | Median | Mean |
| --- | --- | --- | --- | --- |
| Serum | Increased in serum | 1.01 – 578.54 | 1.49 | 7.83 |
|  | Increased in EDTA plasma | 0.11 – 0.98 | 0.9 | 0.83 |
| Heparinized Plasma | Increased in heparin plasma | 1.02 – 40.37 | 1.49 | 2.62 |
|  | Increased in EDTA plasma | 0.08 – 0.98 | 0.9 | 0.84 |
| Citrate Plasma | Increased in citrate plasma | 1.05 – 59.37 | 1.85 | 4.37 |
|  | Increased in EDTA plasma | 0.08 – 0.96 | 0.78 | 0.75 |
| EDTA-anticoagulated whole blood | Increased in whole blood | 1.04 – 11,114 | 2.49 | 55.6 |
|  | Increased in EDTA plasma | 0.0 – 0.99 | 0.57 | 0.58 |
